# *trans*-2,5-Di­methyl­piperazine-1,4-diium dinitrate

**DOI:** 10.1107/S1600536814012100

**Published:** 2014-05-31

**Authors:** Sofian Gatfaoui, Thierry Roisnel, Hassouna Dhaouadi, Houda Marouani

**Affiliations:** a Laboratoire de Chimie des Matériaux, Faculté des Sciences de Bizerte, 7021 Zarzouna Bizerte, Tunisia; b Centre de Diffractométrie X, UMR 6226 CNRS, Unité Sciences Chimiques de Rennes, Université de Rennes I, 263 Avenue du Général Leclerc, 35042 Rennes, France; c Laboratoire des Matériaux Utiles, Institut National de Recherche et d’Analyse Physico-chimique, Pole Technologique de Sidi-Thabet, 2020 Tunis, Tunisia

## Abstract

In the structure of the title salt, C_6_H_16_N_2_^2+^·2NO_3_^−^, the cations are connected to the anions through bifurcated N—H⋯(O,O) and weak C—H⋯O hydrogen bonds, generating corrugated layers parallel to the (100) plane. The organic cation is centrosymmetric and the diprotonated piperazine ring adopts a chair conformation, with the methyl groups occupying equatorial positions.

## Related literature   

For pharmacological properties of piperazine, see: Conrado *et al.* (2008 ▶). For related structures, see: Gatfaoui *et al.* (2013 ▶, 2014*a* ▶,*b* ▶); Marouani *et al.* (2012 ▶); Kefi *et al.* (2013 ▶). For a complex of the title cation, see: Rother *et al.* (1997 ▶). For puckering parameters, see: Cremer & Pople (1975 ▶).
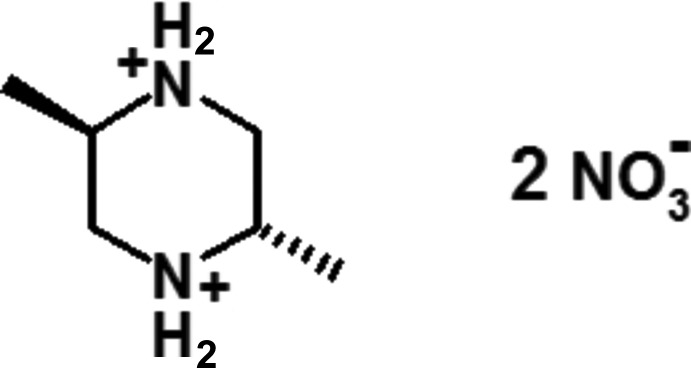


## Experimental   

### 

#### Crystal data  

C_6_H_16_N_2_^2+^·2NO_3_^−^
*M* *_r_* = 240.23
Monoclinic, 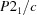

*a* = 7.0357 (8) Å
*b* = 10.0277 (10) Å
*c* = 8.3112 (8) Å
β = 116.149 (8)°
*V* = 526.36 (9) Å^3^
*Z* = 2
Mo *K*α radiation
μ = 0.13 mm^−1^
*T* = 150 K
0.58 × 0.46 × 0.23 mm

#### Data collection  

Bruker APEXII diffractometer
Absorption correction: multi-scan (*SADABS*; Bruker, 2006 ▶) *T*_min_ = 0.827, *T*_max_ = 0.970
4126 measured reflections
1195 independent reflections
1059 reflections with *I* > 2σ(*I*)
*R*_int_ = 0.022

#### Refinement  

*R*[*F* ^2^ > 2σ(*F* ^2^)] = 0.034
*wR*(*F* ^2^) = 0.090
*S* = 1.11
1195 reflections
74 parameters
H-atom parameters constrained
Δρ_max_ = 0.30 e Å^−3^
Δρ_min_ = −0.23 e Å^−3^

### 

Data collection: *APEX2* (Bruker, 2006 ▶); cell refinement: *SAINT* (Bruker, 2006 ▶); data reduction: *SAINT*; program(s) used to solve structure: *SIR97* (Altomare *et al.*, 1999 ▶); program(s) used to refine structure: *SHELXL97* (Sheldrick, 2008 ▶); molecular graphics: *ORTEP-3 for Windows* (Farrugia, 2012 ▶) and *DIAMOND* (Brandenburg & Putz 2005 ▶); software used to prepare material for publication: *WinGX* (Farrugia, 2012 ▶) and *CRYSCAL* (T. Roisnel, local program).

## Supplementary Material

Crystal structure: contains datablock(s) I. DOI: 10.1107/S1600536814012100/bg2529sup1.cif

Structure factors: contains datablock(s) I. DOI: 10.1107/S1600536814012100/bg2529Isup2.hkl

Supporting information file. DOI: 10.1107/S1600536814012100/bg2529Isup3.cml

CCDC reference: 1005191

Additional supporting information:  crystallographic information; 3D view; checkCIF report

## Figures and Tables

**Table 1:** Hydrogen-bond geometry (Å, °)

*D*—H⋯*A*	*D*—H	H⋯*A*	*D*⋯*A*	*D*—H⋯*A*
N2—H2*A*⋯O1^i^	0.90	1.99	2.8471 (14)	158
N2—H2*A*⋯O2^ii^	0.90	2.45	2.9899 (13)	119
N2—H2*B*⋯O1	0.90	2.07	2.9057 (13)	153
N2—H2*B*⋯O3	0.90	2.42	3.2172 (14)	149
C1—H1⋯O1^iii^	0.98	2.50	3.2614 (14)	134

Symmetry codes: (i) 
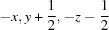
; (ii) 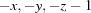; (iii) 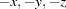.

**Fig. 1.**
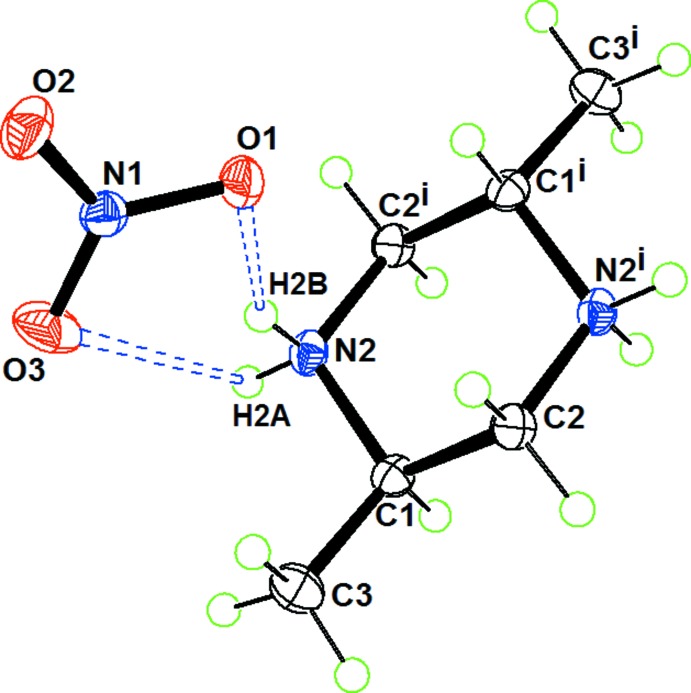
An ORTEP view of (I) with the atom-labelling scheme. Displacement ellipsoids are drawn at the 30% probability level. H atoms are represented as small spheres of arbitrary radii. Hydrogen bonds are shown as dotted lines. Symmetry code: i: -x, -y, -z.

**Fig. 2.**
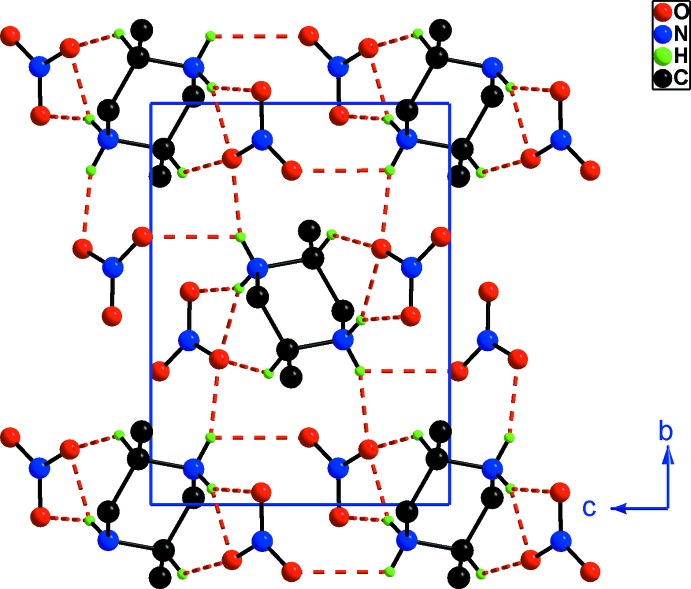
Projection of (I) along the a axis. The H-atoms not involved in H-bonding are omitted.

## supplementary crystallographic information

### 1. Comment

Piperazine and its derivatives are widely used due to their interesting biological and pharmacology proprieties (Conrado *et al.*, 2008). In this work, we report the preparation and the structural investigation of a new organic nitrate, C_6_H_16_N_2_·(NO_3_)_2_ (I). The asymmetric unit of (I) is composed of a half *trans*-2,5-dimeyhylpipeazine-1,4-dium cations and one nitrate anion (Figure 1). In the structure, the cations are connected to the anions through bifurcated N—H···O(O) and weak C—H···O hydrogen bonds, generating a corrugated layers parallel to the (001) plane (Fig. 2).

Interatomic bond lengths and angles of the nitrate anions spread respectively within the ranges [1.2398 (13)–1.2706 (13) Å] and [118.65 (10)–121.73 (10)°]. These geometrical features have also been noticed in other crystal structures (Marouani *et al.*, 2012; Kefi *et al.*, 2013; Gatfaoui *et al.*, 2013, 2014*a*,*b*). It is worth noting that the distance N1—O1 is significantly longer than the N1—O2 and N1—O3 distances because O1 is applied in three hydrogen bonds (table1) while O2 and O3 are applied in only one hydrogen bond. Inside such a structure, the complete organic entity is generated by inversion symmetry located at (0, 0, 0) and (0, 1/2, 1/2). So it is built up by only the half of the cation. Examination of the organic cations shows that the bond distances and angles show no significant difference from those obtained in other complex involving the same organic groups (Rother *et al.*, 1997). The diprotonated piperazine ring adopts a chair conformation, with the methyl groups occupying an equatorial position, with puckering parameters: Q = 0.6083 Å, θ = 90 ° and φ = 166 ° (Cremer & Pople, 1975).

The established H-bonds of types N—H···O(O) and C—H···O involve oxygen atoms of the nitrate anions as acceptors, and protonated nitrogen atoms and methine groups of the *trans*-2,5-dimethylpiperazine-1,4-diium as donors.

### 2. Experimental

An aqueous solution containing 2 mmol of HNO_3_ in 10 ml of water was added to 1 mmol of *trans*-2,5-dimethylpiperazine in 20 ml of water. The obtained solution was stirred for 1 h, filtered and then left to stand at room temperature. Colorless single crystals of the title compound were obtained after some days.

### 3. Refinement

All H atoms were located in a difference map. Nevertheless, they were geometrically placed and refined using a riding model, with C—H = 0.97 Å (methylene), or 0.96 Å (methyl), or 0.98 Å (methine), N—H = 0.90 Å (NH_2_) with *U*_iso_(H) = 1.2*U*_eq_(C or N).

### Crystal data

**Table:**

C_6_H_16_N_2_^2+^·2NO_3_^−^	*F*(000) = 256
*M**_r_* = 240.23	*D*_x_ = 1.516 Mg m^−^^3^
Monoclinic, *P*2_1_/*c*	Mo *K*α radiation, λ = 0.71073 Å
Hall symbol: -P 2ybc	Cell parameters from 2153 reflections
*a* = 7.0357 (8) Å	θ = 3.4–27.4°
*b* = 10.0277 (10) Å	µ = 0.13 mm^−^^1^
*c* = 8.3112 (8) Å	*T* = 150 K
β = 116.149 (8)°	Prism, colourless
*V* = 526.36 (9) Å^3^	0.58 × 0.46 × 0.23 mm
*Z* = 2	

### Data collection

**Table:**

Bruker APEXII diffractometer	1059 reflections with *I* > 2σ(*I*)
Graphite monochromator	*R*_int_ = 0.022
CCD rotation images, thin slices scans	θ_max_ = 27.5°, θ_min_ = 3.4°
Absorption correction: multi-scan (*SADABS*; Bruker, 2006)	*h* = −8→9
*T*_min_ = 0.827, *T*_max_ = 0.970	*k* = −9→12
4126 measured reflections	*l* = −10→10
1195 independent reflections	

### Refinement

**Table:**

Refinement on *F*^2^	Primary atom site location: structure-invariant direct methods
Least-squares matrix: full	Secondary atom site location: difference Fourier map
*R*[*F*^2^ > 2σ(*F*^2^)] = 0.034	Hydrogen site location: inferred from neighbouring sites
*wR*(*F*^2^) = 0.090	H-atom parameters constrained
*S* = 1.11	*w* = 1/[σ^2^(*F*_o_^2^) + (0.0438*P*)^2^ + 0.1423*P*] where *P* = (*F*_o_^2^ + 2*F*_c_^2^)/3
1195 reflections	(Δ/σ)_max_ < 0.001
74 parameters	Δρ_max_ = 0.30 e Å^−^^3^
0 restraints	Δρ_min_ = −0.23 e Å^−^^3^

### Special details

**Table:**

Geometry. All e.s.d.'s (except the e.s.d. in the dihedral angle between two l.s. planes) are estimated using the full covariance matrix. The cell e.s.d.'s are taken into account individually in the estimation of e.s.d.'s in distances, angles and torsion angles; correlations between e.s.d.'s in cell parameters are only used when they are defined by crystal symmetry. An approximate (isotropic) treatment of cell e.s.d.'s is used for estimating e.s.d.'s involving l.s. planes.
Refinement. Refinement of *F*^2^ against ALL reflections. The weighted *R*-factor *wR* and goodness of fit *S* are based on *F*^2^, conventional *R*-factors *R* are based on *F*, with *F* set to zero for negative *F*^2^. The threshold expression of *F*^2^ > σ(*F*^2^) is used only for calculating *R*-factors(gt) *etc*. and is not relevant to the choice of reflections for refinement. *R*-factors based on *F*^2^ are statistically about twice as large as those based on *F*, and *R*- factors based on ALL data will be even larger.

### Fractional atomic coordinates and isotropic or equivalent isotropic displacement parameters (Å^2^)

**Table:**

	*x*	*y*	*z*	*U*_iso_*/*U*_eq_	
N1	0.20352 (15)	−0.09103 (10)	−0.37280 (12)	0.0173 (2)	
O1	0.12714 (14)	−0.13760 (9)	−0.27150 (11)	0.0218 (2)	
O2	0.24377 (15)	−0.16754 (10)	−0.47090 (11)	0.0255 (2)	
O3	0.23709 (16)	0.03059 (9)	−0.36962 (13)	0.0312 (3)	
N2	−0.02504 (15)	0.08866 (10)	−0.14298 (12)	0.0169 (2)	
H2A	−0.0683	0.1671	−0.2001	0.020*	
H2B	0.0282	0.0397	−0.2044	0.020*	
C1	0.14685 (18)	0.11399 (12)	0.04232 (15)	0.0165 (3)	
H1	0.0915	0.1730	0.1057	0.020*	
C2	0.21154 (17)	−0.01747 (12)	0.14274 (15)	0.0176 (3)	
H2C	0.2778	−0.0736	0.0869	0.021*	
H2D	0.3146	−0.0007	0.2653	0.021*	
C3	0.33291 (19)	0.18189 (13)	0.03060 (17)	0.0224 (3)	
H3A	0.3930	0.1230	−0.0257	0.034*	
H3B	0.4381	0.2036	0.1490	0.034*	
H3C	0.2854	0.2621	−0.0389	0.034*	

### Atomic displacement parameters (Å^2^)

**Table:**

	*U*^11^	*U*^22^	*U*^33^	*U*^12^	*U*^13^	*U*^23^
N1	0.0184 (5)	0.0158 (5)	0.0155 (5)	0.0005 (4)	0.0054 (4)	0.0005 (4)
O1	0.0288 (5)	0.0212 (5)	0.0202 (4)	−0.0019 (3)	0.0153 (4)	−0.0007 (3)
O2	0.0331 (5)	0.0274 (5)	0.0203 (4)	0.0033 (4)	0.0158 (4)	−0.0025 (4)
O3	0.0356 (5)	0.0134 (5)	0.0400 (6)	−0.0040 (4)	0.0125 (4)	0.0023 (4)
N2	0.0199 (5)	0.0162 (5)	0.0150 (5)	0.0030 (4)	0.0081 (4)	0.0023 (4)
C1	0.0185 (5)	0.0144 (6)	0.0161 (5)	0.0018 (4)	0.0071 (4)	−0.0010 (4)
C2	0.0166 (5)	0.0168 (6)	0.0178 (5)	0.0020 (4)	0.0062 (4)	0.0016 (4)
C3	0.0207 (6)	0.0178 (6)	0.0287 (6)	0.0003 (5)	0.0112 (5)	0.0009 (5)

### Geometric parameters (Å, º)

**Table:**

N1—O2	1.2398 (13)	C1—C2	1.5188 (17)
N1—O3	1.2403 (14)	C1—H1	0.9800
N1—O1	1.2706 (13)	C2—N2^i^	1.4945 (15)
N2—C2^i^	1.4945 (15)	C2—H2C	0.9700
N2—C1	1.5024 (14)	C2—H2D	0.9700
N2—H2A	0.9000	C3—H3A	0.9600
N2—H2B	0.9000	C3—H3B	0.9600
C1—C3	1.5163 (17)	C3—H3C	0.9600
			
O2—N1—O3	121.73 (10)	C2—C1—H1	108.8
O2—N1—O1	119.62 (10)	N2^i^—C2—C1	111.39 (9)
O3—N1—O1	118.65 (10)	N2^i^—C2—H2C	109.4
C2^i^—N2—C1	112.95 (9)	C1—C2—H2C	109.4
C2^i^—N2—H2A	109.0	N2^i^—C2—H2D	109.4
C1—N2—H2A	109.0	C1—C2—H2D	109.4
C2^i^—N2—H2B	109.0	H2C—C2—H2D	108.0
C1—N2—H2B	109.0	C1—C3—H3A	109.5
H2A—N2—H2B	107.8	C1—C3—H3B	109.5
N2—C1—C3	109.74 (9)	H3A—C3—H3B	109.5
N2—C1—C2	109.11 (9)	C1—C3—H3C	109.5
C3—C1—C2	111.53 (10)	H3A—C3—H3C	109.5
N2—C1—H1	108.8	H3B—C3—H3C	109.5
C3—C1—H1	108.8		

Symmetry code: (i) −*x*, −*y*, −*z*.

### Hydrogen-bond geometry (Å, º)

**Table:**

*D*—H···*A*	*D*—H	H···*A*	*D*···*A*	*D*—H···*A*
N2—H2*A*···O1^ii^	0.90	1.99	2.8471 (14)	158
N2—H2*A*···O2^iii^	0.90	2.45	2.9899 (13)	119
N2—H2*B*···O1	0.90	2.07	2.9057 (13)	153
N2—H2*B*···O3	0.90	2.42	3.2172 (14)	149
C1—H1···O1^i^	0.98	2.50	3.2614 (14)	134

Symmetry codes: (i) −*x*, −*y*, −*z*; (ii) −*x*, *y*+1/2, −*z*−1/2; (iii) −*x*, −*y*, −*z*−1.
